# A Rare Case of Solitary Fibrous Tumor Originating in the Peritoneum: Clinical and Histopathological Insights

**DOI:** 10.7759/cureus.72082

**Published:** 2024-10-21

**Authors:** Adhithya N Balaji, Balaji Balasubramanian, Bushra A Tarawneh

**Affiliations:** 1 Internal Medicine, Università Cattolica Del Sacro Cuore, Rome, ITA; 2 Surgery and Oncology, NMC Specialty Hospital, Abu Dhabi, ARE; 3 Pathology, NMC Royal Hospital, Khalifa City, Abu Dhabi, ARE

**Keywords:** abdominal mass, case report, cd34, gist, immunohistochemistry, peritoneum, solitary fibrous tumor, stat6, surgical resection

## Abstract

Solitary fibrous tumors (SFTs) are rare mesenchymal neoplasms that most commonly arise from the pleura, with peritoneal origin being exceptionally uncommon. We report a case of a 51-year-old male who presented with a large abdominal mass, subsequently diagnosed as a solitary fibrous tumor originating from the peritoneum. Radiological evaluation revealed a well-defined mass with a vascular pedicle supplied by the inferior mesenteric artery, extending from the pelvis to the lower abdomen. Surgical resection of the tumor, including part of the rectosigmoid junction was performed and histopathology confirmed the diagnosis of SFT.

Immunohistochemistry (IHC) demonstrated positive staining for CD34, BCL2, and STAT6, supporting the diagnosis. The patient remains disease-free after four years of follow-up. This case contributes to the limited literature on peritoneal SFTs, highlighting the importance of IHC in clinching the diagnosis and of long-term follow-up due to the potential for late recurrence.

## Introduction

Solitary fibrous tumors (SFTs) are rare type of spindle cell tumors of mesenchymal origin, most frequently affecting the thoracic pleura [1]. SFTs account for less than 2% of all soft tissue tumors. They are usually benign and asymptomatic, sometimes detected due to the pressure effect on the adjacent organs. Imaging is done with either computerized tomography (CT) or magnetized resonance tomography (MRI), which assists in predicting the possibility of an SFT. Ideally, these cases are treated with complete surgical resection. It needs long term follow-up for 20 years as recurrences up to two decades have been documented [2].

Due to the rarity of SFTs in the peritoneum, little is known about the biological behavior and optimal management in this location. In this report, we present a case of a 51-year-old man who presented with a swelling in the abdomen and was diagnosed with SFT originating from the peritoneum, detailing its clinical presentation, histopathological findings, management approach and contributing to the limited literature on this unusual entity.

## Case presentation

A 51-year-old male was evaluated for a swelling in the lower abdomen. Clinically, the patient was well built, with truncal obesity. The patient showed no pallor, jaundice, lymphadenopathy or any signs of hemodynamic instability, and was asymptomatic without any symptoms of bowel obstruction. Physical examination showed a large pelvic mass felt in the lower abdomen. It was mobile in the transverse axis and a left-sided direct inguinal hernia was present. He had an ultrasound (US) scan followed by a contrast computerized tomography (CT) scan of the abdomen.

Radiological findings

**Figure 1:**
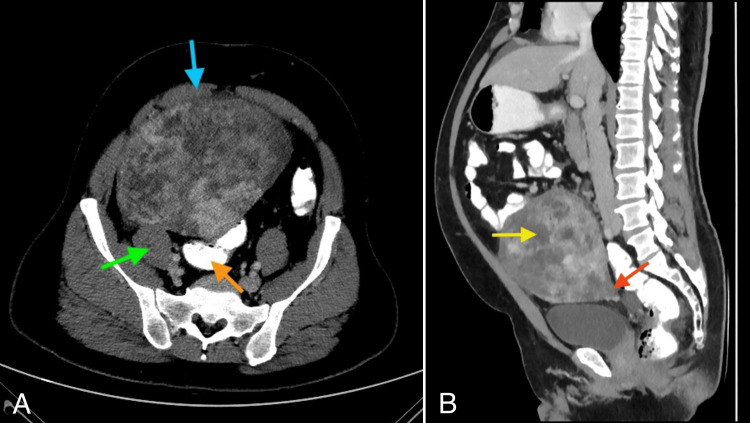
Computerized Tomography (CT) scan of the abdomen – axial section (A) and sagittal section (B). Axial section shows the tumor indenting the sigmoid colon posteriorly (orange arrow), psoas muscle posteriorly (green arrow), and abdomen wall anteriorly (blue arrow). Sagittal section shows the lower end of the mass (red arrow) supplied by branches from the inferior mesenteric artery through a pedicle near rectovesical region. A non-enhancing necrotic area (yellow arrow) is also noted.

The CT scan revealed a large, well-defined oval mass lesion measuring 16 cm x 15 cm x 12 cm in the central and right pelvic region, extending superiorly into the abdomen, predominantly on the right side. A vascular pedicle was seen along the base of the mass in the perirectal region. The arterial supply of the mass was through the tortuous inferior mesenteric artery branch which enters the mass through the base in the rectovesical region. The mass showed heterogenous contrast enhancement with few internal non-enhancing necrotic areas (Figure 1B). Anteriorly, the mass was abutting the inner aspect of the lower abdominopelvic wall and superiorly, it was extending up to the lower pole of the left kidney. The mass was displacing the small bowel loops towards the periphery and a few bowel loops were seen abutting the superior and lateral aspect of the mass. Posteriorly, the mass was indenting on the sigmoid colon and on the right side it was indenting the anterior surface of the psoas muscle (Figure 1A). Possible differential diagnosis included sarcoma, gastrointestinal stromal tumor (GIST), lymphoma, and desmoid tumor. An incidental small, calcified nodule was noticed in the right lower zone of the lung as well as left inguinal cystocele was seen.

Further evaluation was done with the CT chest. It showed a benign-appearing nodule, possibly a healed granulomatous calcified lesion in the right lung base. 

Other workup

His blood investigations showed normal tumor marker levels including alpha-fetoprotein (AFP), human chorionic gonadotropin (beta hCG) and carcinoembryogenic antigen (CEA). Complete blood count (CBC), kidney and liver function tests were unremarkable. He had a colonoscopy which showed no abnormality in the colonic mucosa. There was an extrinsic mass effect seen in upper rectum, but the mucosa was found to be intact.

The case was discussed in the multidisciplinary tumor board (MDT) meeting and a plan was made to proceed with explorative surgery and resection of the mass. A possibility of GIST from the rectal wall was thought as possible differential diagnosis due to the proximity to the rectal wall and the pedicle which was arising from the inferior mesenteric artery.

Surgical findings

**Figure 2:**
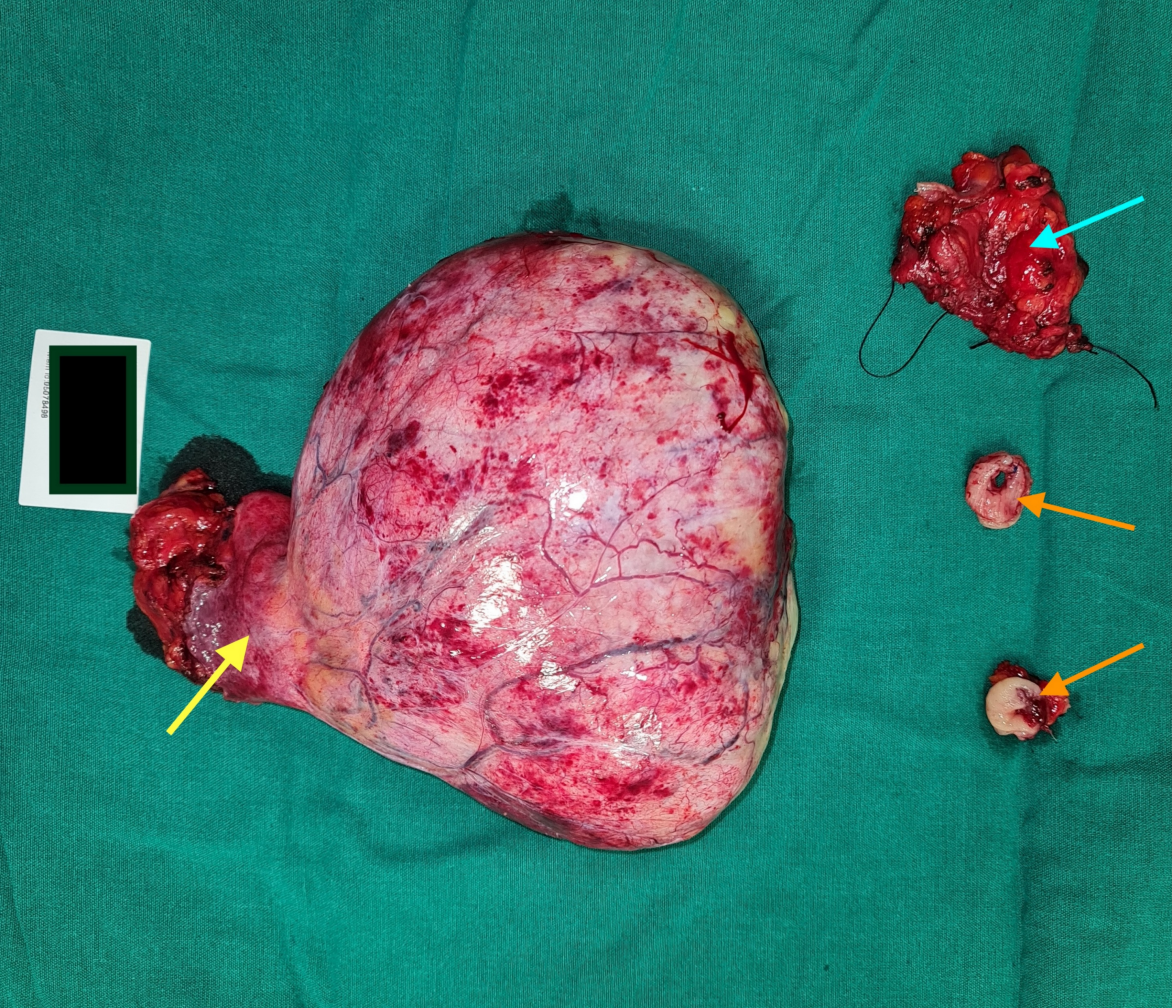
Surgical specimen from laparotomy showing the base which had the main blood supply (yellow arrow), the rectal segment (blue arrow), the proximal and distal doughnut from staplers (orange arrows).

During the laparotomy, a large vascular and encapsulated tumor was found in the abdomen, originating from the pelvis. In the abdomen, it was adherent to the small bowel mesentery and in the pelvis, it was extending to the perirectal tissue, with proximity to the rectal wall. The tumor was resected completely without spill along with the rectosigmoid segment where there was a doubt of origin of the tumor (Figure 2). A 7 cm segment around the rectosigmoid junction was resected, and the distal sigmoid colon was primarily anastomosed to the mid-rectum. The patient had a smooth recovery. He passed stools on the third postoperative day and was started on liquid diet, and was discharged on the fifth postoperative day on a full oral diet.

Histopathological findings

Gross Findings

Histopathology showed a single polypoidal lesion weighing 1001 g. The pedicle was formed by possible mesenteric fact. The surface was intact with congested vessels. The lesion was measuring 16.5 cm x 14 cm x 7 cm, and the pedicle was measuring 3.5 cm x 5 cm x 3.5 cm. The cut section showed a grey-white, firm, multinodular lesion with multiple cystic spaces, hemorrhage, and myxoid changes. The rectum was found to be normal. The doughnut was found to be free of tumor.

Microscopical Findings

**Figure 3:**
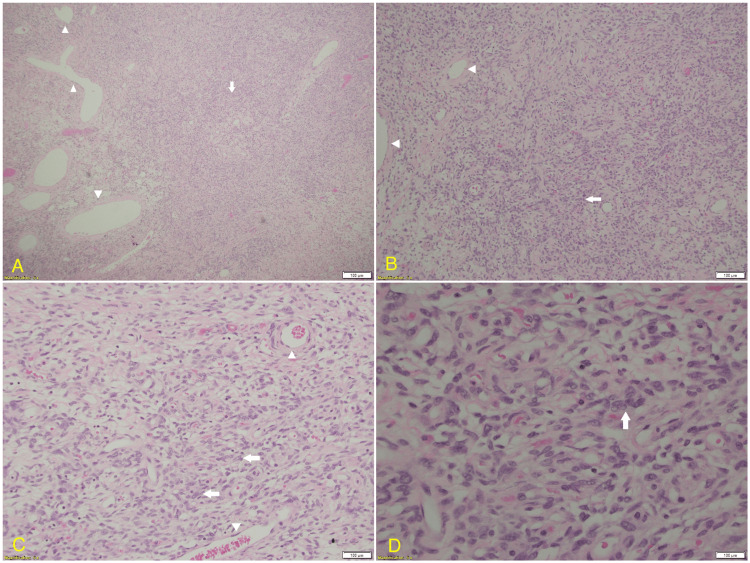
Histologic sections stained with H&E demonstrate a moderately cellular neoplasm with spindle cells arranged in a patternless architecture with hypo- and hypercellular areas in a collagenous stroma with prominent dilated staghorn type vasculature (arrowheads). Individual cells (arrows) are bland and uniform oval to spindle cells dispersed along with thin parallel collagen bands. No atypia, mitotic figures or necrosis was seen. A – 40x magnification, B – 100x magnification, C – 200x magnification, D – 400x magnification H&E – Hematoxylin and eosin

Microscopy showed unencapsulated neoplasm with cells arranged in patternless architecture of hypo and hypercellular areas separated by thick, hyalinized collagen with cracking artifact and staghorn vessels. Individual cells were bland and uniform, and oval to spindle cells were dispersed along with thin parallel collagen bands. Cells had minimal cytoplasm with small, elongated nuclei and indistinct nucleoli. Focal myxoid change, mast cells and adipose tissue were observed along with perivascular sclerosis. Hyalinized and edematous stroma with focal perivascular arrangement was noted. Congested blood vessels, hemorrhage and lymphocytic infiltration were also noted in the periphery (Figure 3). No atypia, mitotic figures or necrosis was seen.

Immunohistochemistry (IHC)

**Figure 4:**
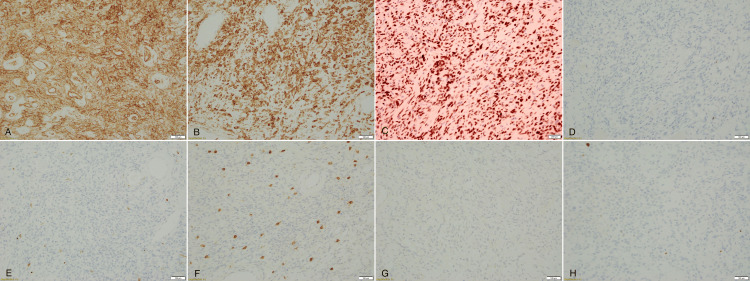
Immunohistochemistry panel of the specimen revealed the tumor cells are positive for CD34 (A), BCL2 (B) and STAT6 (C), and negative for S100 (D), Desmin (E), CD117 (F) and DOG-1 (G). Ki67 (H) is less than 1%. STAT6 - Signal Transducer and Activator of Transcription 6

Immunohistochemical staining confirmed the diagnosis of SFT through strong positivity for CD34, BCL2, and STAT6 (Figures 4A, 4B, 4C). CD34, a mesenchymal marker, is commonly expressed in SFTs but can also appear in other soft tissue tumors, while BCL2 supports the diagnosis, though it lacks specificity. STAT6, the most specific marker for SFTs, results from the NAB2-STAT6 gene fusion and is considered pathognomonic for SFTs [1]. The tumor was negative for S100 (Figure 4D), Desmin (Figure 4E), CD117 (Figure 4F), and DOG1 (Figure 4G), ruling out differential diagnoses like schwannoma, leiomyosarcoma, and GIST. The low Ki-67 index (2%) (Figure 4H) suggested low malignant potential.

The case was re-discussed in the MDT, and it was decided to keep the patient under close follow up. He is undergoing regular annual CT scans and over the past four years, he is free of disease recurrence. 

## Discussion

SFTs are rare mesenchymal tumors documented in literature. Most commonly these lesions arise from pleura [1]. SFT was first described by Klemperer and Rabin as a pleural neoplasm in 1931 [3]. Few cases have been documented in the abdomen, especially those arising from the mesentery. Other rare sites include omentum where six cases have been reported in literature [4]. SFTs of mesenteric peritoneum occur in both genders and in all ages, but predominantly affect males between the fourth and seventh decades of life, with a median age of 54 [1,5,6].

Most SFTs are asymptomatic and are diagnosed either incidentally or when they cause pressure symptoms due to the compression of the adjacent organs.

With regards to imaging, CT and MRI scans are effective tools in diagnosing SFTs [7]. Most of these tumors exhibit a round or lobulated shape with distinct boundaries, often growing into large, fully encapsulated masses [7]. On CT scans, they typically present as multiloculated soft tissue masses with varied enhancement and areas of calcification. Calcifications may appear densely packed or scattered, and central regions with low attenuation, often tubular or rounded, point to areas of necrosis [1]. Enhancement is most pronounced during the arterial and early portal venous phases, with a reduction in contrast during the delayed phase. However, if the tumor is predominantly fibrous, delayed-phase imaging shows more prominent contrast enhancement [5].

Differential diagnosis of SFTs include desmoid tumor, GIST, inflammatory pseudotumor, fibromatosis and leiomyoma. The presence of necrotic areas, calcifications and enhancement in the arterial and early venous phase, with complete washout in the delayed phase point towards the diagnosis of SFT [1]. Few of the sarcomas need to be considered as differential diagnosis like fibrosarcoma, malignant fibrous histiocytoma, leiomyosarcoma, hemangiopericytoma, synovial sarcoma, and malignant mesenchymomas [8].

As in our case, most of the cases were treated surgically. Surgery is the cornerstone for curative treatment, while chemoradiation is only utilized in non resectable tumors [9]. There is no documented role for other treatments like chemotherapy or radiotherapy. Few case reports have demonstrated the effectiveness of radiotherapy in the control of SFTs. But there is no conclusive evidence to support radiotherapy or chemotherapy in SFTs [10].

The diagnosis of SFT is made by the pathologist with the aid of IHC. SFTs develop from CD34-positive dendritic mesenchymal cells, which have the potential to differentiate into various cell types, including fibroblasts, myofibroblasts, and vascular epithelial cells [9]. Latest IHC study includes CD34, CD99, BCL2 and STAT6 which are the most useful positive IHC markers for SFTs [11]. Among these, the expression of STAT6 in the tumor cell is essential for diagnosis when CD34 and CD99 are negative. In IHC, the positivity of CD34 and STAT 6, is regarded as reliable diagnostic marker [6].

Originally classified as hemangiopericytomas, the WHO has updated the terminology in 2016, changing the nomenclature to SFT [12]. Most SFTs are benign, although a few cases of malignant SFTs have been reported [8,9]. Malignancy is defined by the degree of local invasion, recurrence and distant metastases. Microscopically, SFTs are classified as either benign or malignant based on histological features, such as high cellularity, mitotic activity, pleomorphism, hemorrhage, and necrosis [8]. Numerous studies have identified nuclear atypia, a high mitotic count (>4/10 high power fields), tumor size greater than 10 cm, positive surgical margins, and the presence of necrosis and hemorrhage as the most reliable predictors of poor prognosis [9]. In a study by Demicco et al., it was observed that older patients with tumors larger than 15 cm have an elevated risk of metastasis [8]. Approximately 15% to 20% of SFTs are malignant, particularly those with a size greater than 10 cm [8]. For abdominopelvic SFTs exceeding 20 cm, complete surgical resection has been shown to significantly reduce the likelihood of local recurrence and distant metastasis, which can occur in up to 8% of cases [8].

A study by O'Neill et al. showed that the lungs were the most common site of metastasis, occurring in 61% of cases, followed by the pleura (49%), and then the liver, bones, and peritoneum, each affecting 41% of cases. A significantly higher percentage of patients with extra-thoracic SFT developed metastases (27%) compared to those with thoracic SFT (9%) (p = 0.003). The median metastasis-free survival for patients with extra-thoracic SFT was 117 months, while it was 120 months for those with thoracic SFT (p = 0.01) [13].

## Conclusions

SFTs typically present as incidental tumors and rarely cause symptoms due to the compression of adjacent organs. Imaging with CT or MRI might give a clue in the possible diagnosis, although it may not be confirmatory. IHC is essential to clinch the diagnosis with CD34 and STAT 6 being the most specific markers. Although most of them are benign, some tumors are defined as malignant based on the histological characteristics. The essential treatment includes complete surgical removal with a clear margin. Recurrences have been documented even after extended periods, underscoring the importance of long-term follow-up. A follow-up duration of 20 years is therefore strongly recommended for optimal monitoring.
